# GVPC Medium Manufactured without Oxygen Improves the Growth of *Legionella* spp. and Exhibits Enhanced Selectivity Properties

**DOI:** 10.1128/spectrum.02401-21

**Published:** 2022-03-22

**Authors:** Pablo Casino, Asunción López, Sara Peiró, Martín Ríos, Santiago Ríos, Aldous Porta, Gemma Agustí, Daniel Asensio, Ana María Marqués, Núria Piqué

**Affiliations:** a Department of Quality Control, Reactivos para Diagnóstico, S.L., Barcelona, Catalonia, Spain; b Microbiology Section, Department of Biology, Healthcare and Environment, Faculty of Pharmacy and Food Sciences, Universitat de Barcelona, Barcelona, Catalonia, Spain; c Department of Genetics, Microbiology and Statistics, Biology Faculty, Universitat de Barcelona, Barcelona, Catalonia, Spain; d Department of Biochemistry and Biothecnology, Human Nutrition Unit, Universitat Rovira i Virgili, Reus, Catalonia, Spain; e Institut de Recerca en Nutrició i Seguretat Alimentària de la UB (INSA-UB), Universitat de Barcelona, Barcelona, Catalonia, Spain; University of Minnesota

**Keywords:** *Legionella pneumophila*, *Legionella anisa*, GVPC, recovery, selectivity, oxygen, *Pseudomonas aeruginosa*, *Enterococcus faecalis*

## Abstract

Glycine-vancomycin-polymyxin-cycloheximide agar (GVPC) is a recommended medium for the detection of *Legionella* spp. in water samples. However, its quality could be improved in terms of recovery of *Legionella* spp. and selectivity properties. Modifications were introduced in GVPC manufacture: autoclaving conditions (115°C, 15 min) and atmosphere during component-stirring (removal of oxygen and N_2_ injection). The use of softer autoclaving conditions (115°C, 15 min) improved the growth of Legionella anisa by the spiral method and Legionella pneumophila after membrane filtration. The medium manufactured with O_2_ removal and autoclaving for 15 min at 115°C allowed a faster growth of L. pneumophila (colonies visible at day 2) and a notable increase of L. anisa growth (colonies appearing at day 3, and statistically significant numbers of CFU at day 5). After 3 to 5 days of incubation, the improved media showed higher selectivity properties, particularly for Enterococcus faecalis ATCC 29212 and Pseudomonas aeruginosa ATCC 9027. A further improvement was achieved by the addition of N_2_ during ingredient stirring, leading to a statistically significant faster growth of L. pneumophila at days 2 and 3 and L. anisa at day 3. Selectivity properties were also enhanced, resulting in the complete inhibition of both E. faecalis strains and Escherichia coli and complete-partial inhibition of P. aeruginosa. Oxygen removal during GVPC manufacture using a vacuum pump system promotes the growth of L. pneumophila and L. anisa, and markedly inhibits the growth of E. coli, P. aeruginosa, and E. faecalis.

**IMPORTANCE** Currently, GVPC is a recommended medium for the detection of *Legionella* spp. in water samples. However, recovery of *Legionella* spp. and selectivity properties can be improved. GVPC medium manufactured without oxygen improved the growth of Legionella pneumophila and Legionella anisa. Oxygen removal during GVPC manufacture also improved selectivity properties. A further improvement was achieved by the addition of N_2_ during ingredient stirring, leading to a faster growth of L. pneumophila at days 2 and 3 and L. anisa at day 3 and enhancement of selectivity properties. The introduction of the modified GVPC medium in routine practice can allow a better detection of *Legionella* spp. in water samples.

## INTRODUCTION

Legionellosis, the disease transmitted through the inhalation of particles of aerosolized water contaminated by *Legionella* spp., remains an important public health threat (1, 2), with an increasing incidence worldwide in recent years (1–4). Its mortality rate is still high, particularly in immunocompromised patients (1, 3).

The majority of legionellosis outbreaks are correlated with Legionella pneumophila, in particular serogroup 1, although other serogroups and species are also associated with human disease, such as *L. micdadei* (now classified as Tatlockia micdadei), *L. dumoffii*, and *L. longeachae* (3, 5). Any system or equipment containing, storing, or re-circulating non-sterile water that can be aerosolized is a potential source of legionellosis (3, 5), including water systems of hospitals, hotels, private houses, cooling towers, dental units, and recreational or therapeutic facilities (5–12).

*Legionella* testing, which is essential for the prevention and control of legionellosis outbreaks, represents a constant challenge at an environmental and clinical level (13–15). Culture and isolation in selective media are the standard methods to confirm Legionella pneumophila infection in clinical samples (13) and to detect L. pneumophila in water samples, despite requiring prolonged incubation periods of up to 10 days (4, 16). However, this could change with the recent development and validation of new rapid techniques for the detection of viable *Legionella* (14, 17, 18).

In clinical samples, the most successful procedure includes the use of buffered charcoal yeast extract (BCYE) agar containing 0.1% α-ketoglutarate with l-cysteine incubated at 35°C in a humidified, 2.5% CO_2_ atmosphere (13, 19). Most isolates demonstrate growth in 3 to 5 days, but non-pneumophila *Legionella* species and occasionally primary-specimen isolates may require considerably longer incubation times, sometimes up to 2 weeks (13), thus reinforcing the need for improved culture media that allow a faster growth.

In water samples, the culture plate technique is the standard method stipulated by current regulations and requires more than 10 days to obtain results. In search of a more rapid method, multiple alternatives are currently being developed, including viability qPCR (14) and immunodetection of bacteria retained in nitrocellulose filters (17), with the challenge of distinguishing between dead and live cells (14, 18). According to ISO 11731:2017, water samples with high concentrations of interfering microorganisms require selective media, such as glycine vancomycin polymyxin and cycloheximide agar (GVPC) or the Wadowsky and Yee medium (MWY), whereas low concentrations of interfering microorganisms require the use of selective media, such as GVPC, MWY, or BCYE+AB agar (BCYE containing polymixin B, sodium cefazolin, and pimaricin), together with non-selective media, such as BCYE agar (16).

GVPC, the medium used for the isolation and enumeration of *Legionella* in water samples, is identical to BCYE agar (containing yeast extract, agar, activated charcoal, α-ketoglutarate, ACES buffer, potassium hydroxide, l-cysteine, and iron (III) pyrophosphate) except for the addition of four selective compounds: glycine, polymyxin B sulfate, vancomycin hydrochloride, and cycloheximide (16). In GVPC medium, the presence of activated charcoal has a key role as scavenger of radicals and peroxides, to which the genus *Legionella* is particularly sensible (20–22), while α-ketoglutarate, with antioxidant properties (23), l-cysteine, and iron (III) pyrophosphate are added to stimulate *Legionella* growth.

Although it is one of the media recommended in ISO 11731:217 and one of the most frequently used selective media by laboratories, it has certain shortcomings that greatly affect recovery of *Legionella* spp. and its selectivity properties (24). In particular, it is inefficient in inhibiting the growth of Pseudomonas aeruginosa, which could explain the removal of this parameter (Pseudomonas aeruginosa inhibition) from the quality control requirements of this medium according to ISO 11731:2017. Difficulties in inhibiting the growth of a Enterococcus faecalis strain recommended for quality control by ISO 11133:2014 (E. faecalis ATCC 29212) are also common. This has prompted most GVPC manufacturers to opt for the other recommended strain, E. faecalis ATCC 19433, for the medium quality control. The quality of the culture media used to isolate *Legionella* spp. has a great influence on the process of standardization and quality assurance. However, few studies aimed at improving GVPC medium properties have been published to date.

In this context, the present study was performed to assess the factors in the manufacture of GVPC according to ISO 11731:2017 that may influence the detection of *Legionella* spp. in terms of growth rate, productivity (recovery of L. pneumophila and Legionella anisa) and selectivity (E. faecalis, Escherichia coli, and P. aeruginosa). We found that slight modifications can significantly improve the productivity and selectivity properties of GVPC.

## RESULTS

### Effect of modified autoclaving conditions (115°C, 15 min) on the productivity and selectivity of GVPC (conditions 2 versus conditions 1).

**FIG 1:**
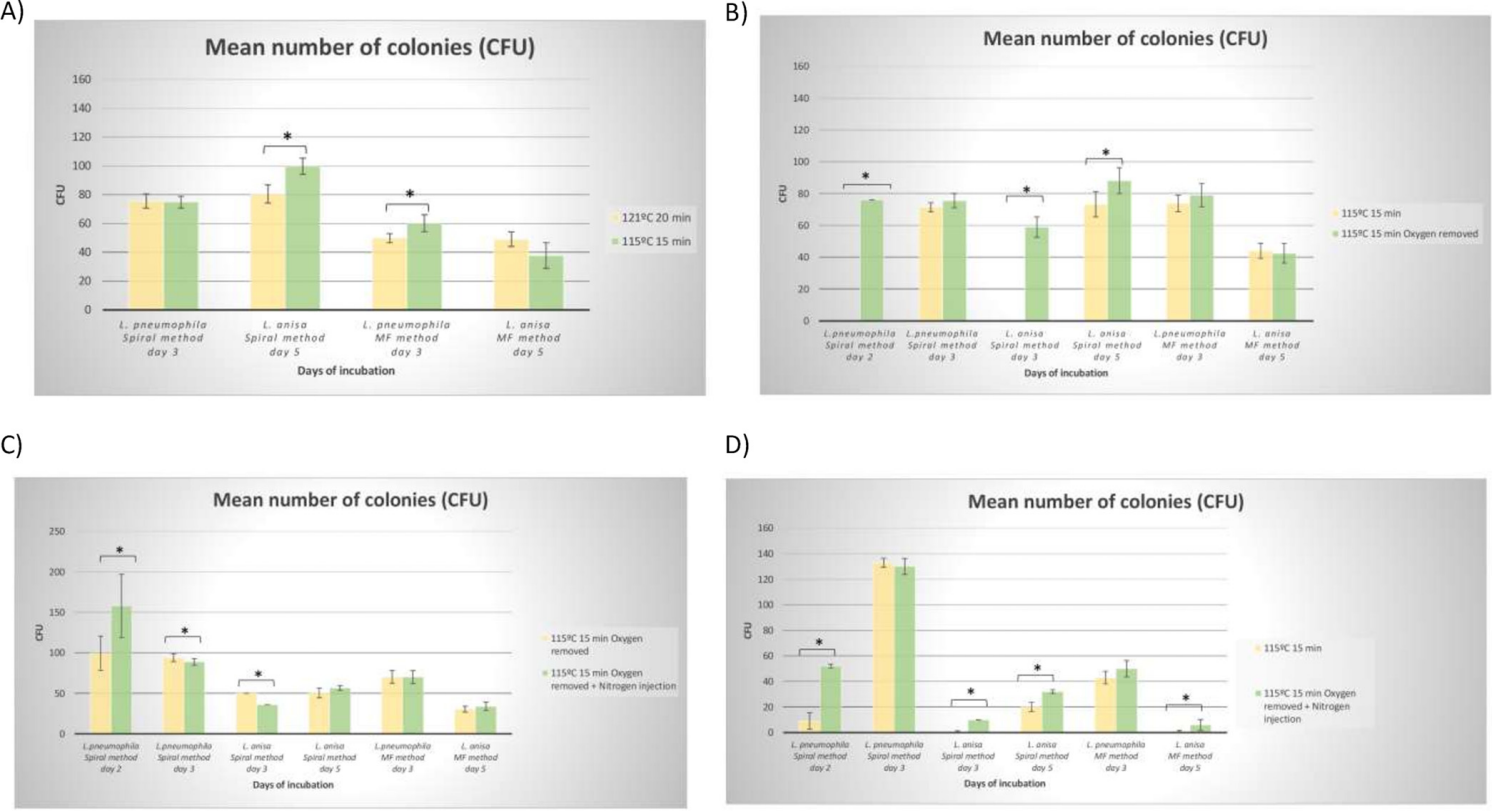
Comparison of growth in GVPC at days 3 and 5 in different conditions. (A) Legionella pneumophila at day 3 (spiral-pour method): normal conditions versus 115°C, 15 min, with O_2_ (conditions 1 versus conditions 2). (B) Legionella pneumophila at day 3 (spiral-pour method): normal conditions versus 115°C, 15 min, without O_2_, N_2_ injection (conditions 1 versus conditions 4). (C) Legionella anisa at day 5 (spiral-pour method): normal conditions versus 115°C, 15 min, without O_2_, N_2_ injection (conditions 1 versus conditions 4). (D) Legionella pneumophila at day 3 (membrane filtration): normal conditions versus 115°C, 15 min, without O_2_ (conditions 1 versus conditions 3). (E) Legionella anisa at day 3 (spiral-pour method): normal conditions versus 115°C, 15 min, without O_2_ (conditions 1 versus conditions 3).

**FIG 2:**
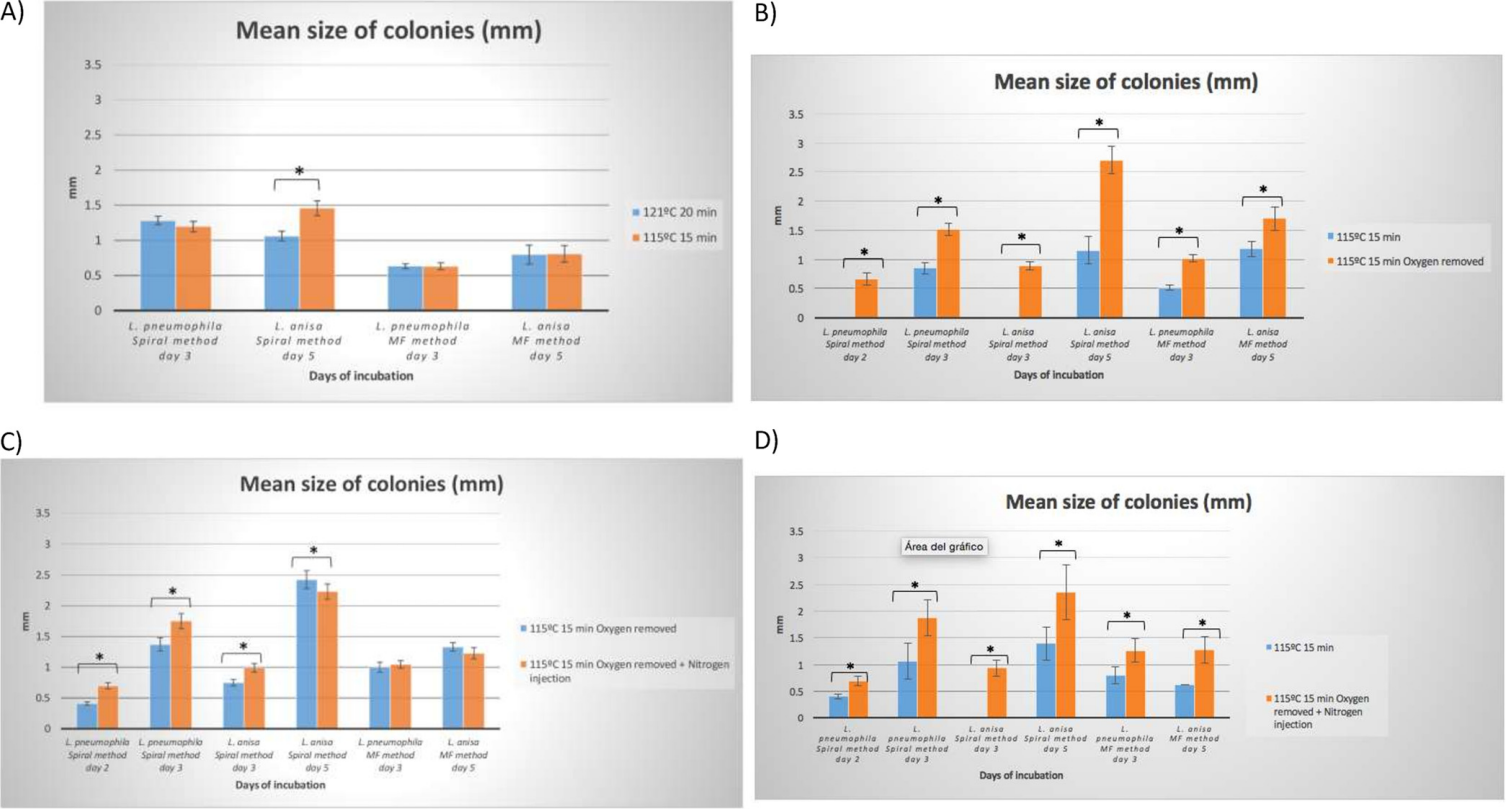
Bar graph showing mean CFU obtained in GVPC manufactured in different conditions for L. pneumophila and L. anisa seeded by the spiral-pour method and membrane filtration (MF). A) Conditions 1 (121°C, 20 min) vs conditions 2 (115°C, 15 min). B) Conditions 2 (115°C, 15 min) vs conditions 3 (115°C, 15 min, without oxygen). C) Conditions 3 (115°C, 15 min, without oxygen) vs conditions 4 (115°C, 15 min, without oxygen, nitrogen injection). D) Conditions 2 (115°C, 15 min) vs conditions 4 (115°C, 15 min, without oxygen, nitrogen injection).

**FIG 3:**
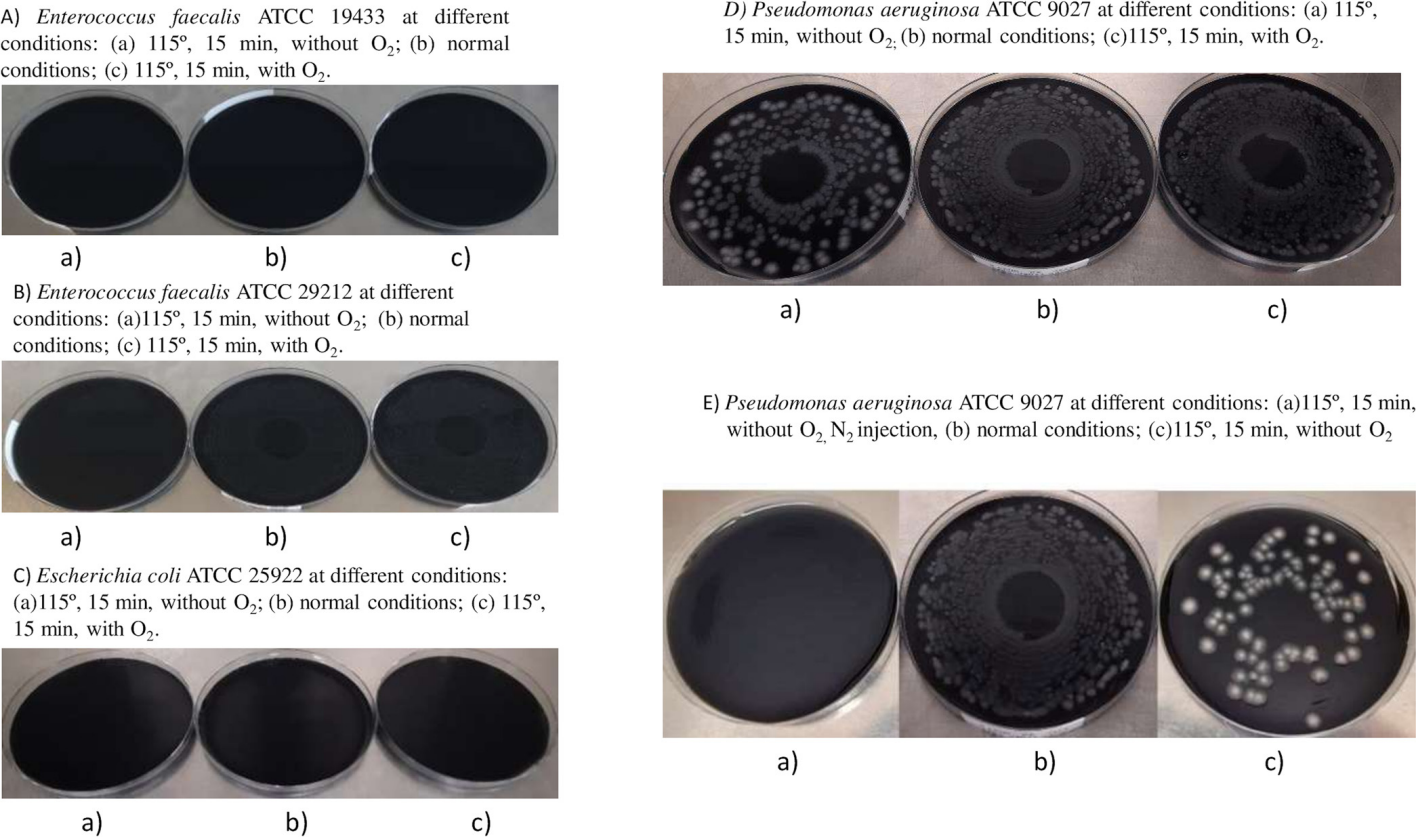
Bar graph showing mean colony size (mm) obtained in GVPC manufactured in different conditions for L. pneumophila and L. anisa seeded by the spiral method and membrane filtration (MF). A) Conditions 1 (121°C, 20 min) vs conditions 2 (115°C, 15 min). B) Conditions 2 (115°C, 15 min) vs conditions 3 (115°C, 15 min, without oxygen). C) Conditions 3 (115°C, 15 min, without oxygen) vs conditions 4 (115°C, 15 min, without oxygen, nitrogen injection). D) Conditions 2 (115°C, 15 min) vs conditions 4 (115°C, 15 min, without oxygen, nitrogen injection).

The media manufactured under different conditions were compared to understand the influence of different factors on growth rate, productivity, and selectivity. In comparison with normal autoclaving conditions (121°C, 20 min), a reduction of 6°C and 5 min in the autoclaving cycle of the thermoresistant ingredients yielded better L. pneumophila and L. anisa growth. Using the spiral-pour method, similar mean numbers of CFU were observed for L. pneumophila at day 3 with conventional and lower autoclaving temperatures (74.75 ± 3.98 CFU versus 75.50 ± 4.49 CFU; *P* = 0.2852; ANOVA test), and mean colony sizes were also similar (1.20 ± 0.075 mm versus 1.28 ± 0.06 mm; *P* = 0.0797, Student’s *t* test for independent data) (Fig. 1A, 2A, and 3A).

For L. pneumophila, a statistically significant increase in the mean number of CFU was observed after membrane filtration (60.12 ± 5.90 at 115°C 15 min versus 49.75 ± 3.18 at 121°C, 20 min; *P* = 0.0327; ANOVA test) at day 3, without differences being observed in the colony size (0.63 mm in both conditions) (Fig. 2A and 3A).

In the case of L. anisa, a notable increase in the mean number of CFU was observed by the spiral-pour method at day 5 (99.62 ± 5.56 versus 80.5 ± 6.36; *P* = 0.0004; ANOVA test), while no improvements in growth were detected after membrane filtration (37.58 ± 8.89 at 115°C 15 min versus 49.00 ± 4.94 under normal conditions; *P* = 0.2737; ANOVA test). Similar results were observed for colony sizes at day 5: 1.46 mm versus 1.06 mm by the spiral-pour method (*P* < 0.001; Student’s *t* test for independent data) and 0.81 mm versus 0.80 mm by membrane filtration (*P* = 0.8556; Student’s *t* test for independent data) (Fig. 2A and 3A).

**FIG 4:**
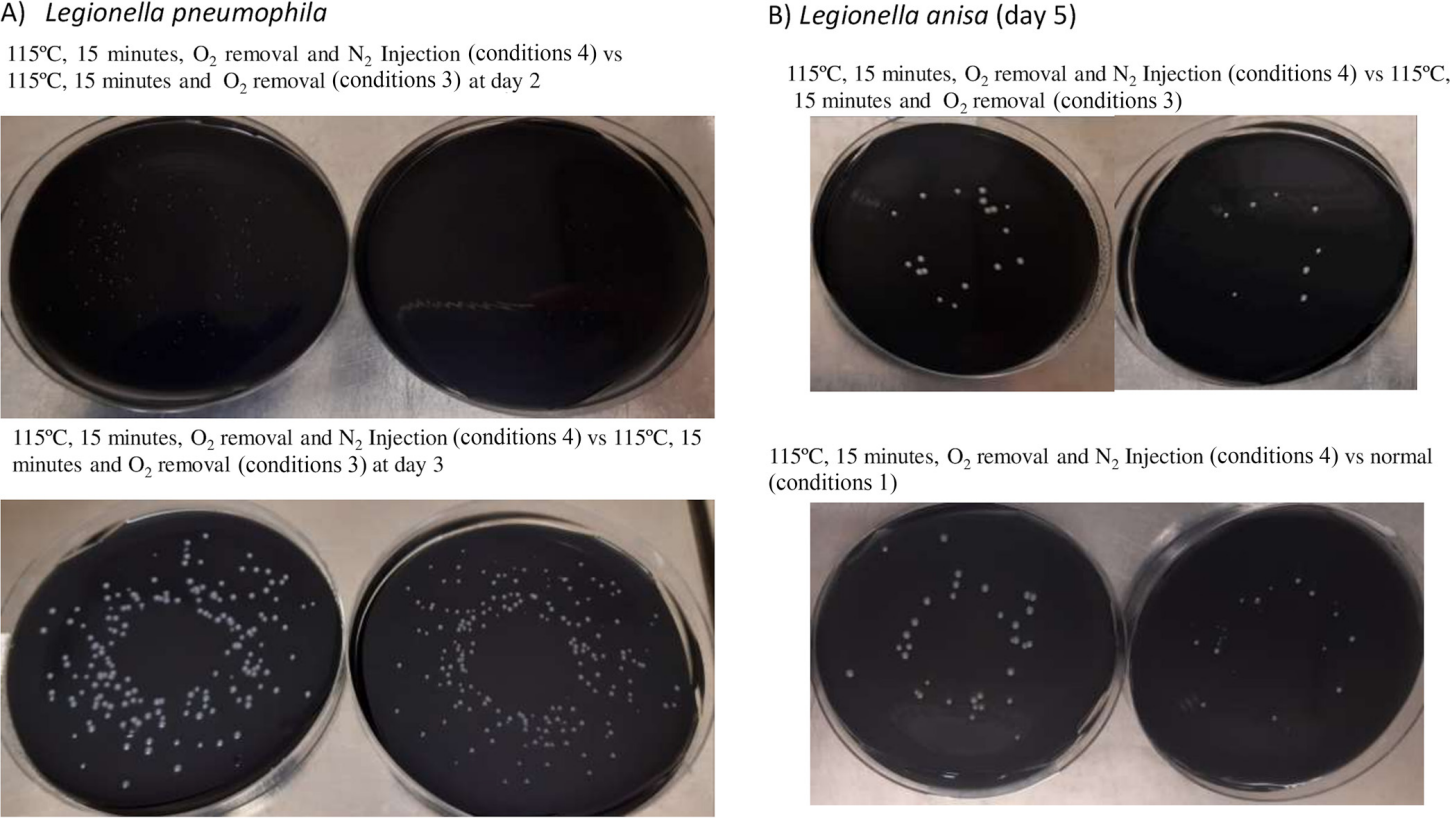
Comparison of growth in GVPC manufactured with O_2_ removal and N_2_ injection during the ingredient mixture at 115°C, 15 min. (A) Enterococcus faecalis ATCC 19433 growth after incubation at 36 ± 2°C for 3 days in GVPC manufactured in different conditions: (a) 115°C, 15 min, without O_2_ (conditions 3); (b) normal conditions (conditions 1); (c) 115°C, 15 min (conditions 2). (B) Enterococcus faecalis ATCC 29212 growth after incubation at 36 ± 2°C for 3 days in GVPC manufactured in different conditions: (a) 115°C, 15 min, without O_2_ (conditions 3); (b) normal conditions (conditions 1); (c) 115°C, 15 min (conditions 2). (C) Escherichia coli ATCC 25922 growth after incubation at 36 ± 2°C for 3 days in GVPC manufactured in different conditions: (a) 115°C, 15 min, without O_2_ (conditions 3); (b) normal conditions (conditions 1); (c) 115°C, 15 min (conditions 2) (D) Pseudomonas aeruginosa ATCC 9027 growth after incubation at 36 ± 2°C for 3 days in GVPC manufactured in different conditions: (a) 115°C, 15 min, without O_2_ (condition 3); (b) normal conditions (conditions 1); (c) 115°C, 15 min (conditions 2). (E) Pseudomonas aeruginosa ATCC 9027 growth after incubation at 36 ± 2°C for 3 days in GVPC manufactured in different conditions: (a) 115°C, 15 min, without O_2_, N_2_ injection (conditions 4); (b) normal conditions (conditions 1); (c) 115°, 15 min, without O_2_ (conditions 3).

Under these conditions, no relevant differences in selectivity properties were observed, assessed by the growth of E. faecalis, P. aeruginosa, and E. coli. Total inhibition was obtained for E. coli ATCC 25922 and E. faecalis ATCC 19433, and a high degree of P. aeruginosa ATCC 9027 and E. faecalis ATCC 29212 growth was observed in both conditions (> 1000 CFU) (Table 1; Fig. 4).

### Effect of O_2_ removal during medium manufacture using a vacuum pump system (conditions 3 versus conditions 2).

Based on the above results, modified autoclaving (115°C for 15 min) was used in the manufacture of media with O_2_ removal.

Oxygen removal and modified autoclaved conditions (conditions 3) produced a higher growth of L. pneumophila and L. anisa than modified autoclaving alone (115°C, 15 min, with O_2_, conditions 2). Notably, the medium produced without O_2_ allowed a faster growth of L. pneumophila, with colonies being visible at day 2, particularly when using the spiral-pour method (76 CFU versus 0 CFU). Moreover, at day 3, a non-significant increase was observed in the growth of L. pneumophila assessed as a mean number of CFU by the spiral-pour method (75.57 ± 4.49 CFU versus 71.50 ± 2.82 CFU; *P* = 0.8528; ANOVA test) and after membrane filtration (79.03 ± 7.30 versus 73.83 ± 5.18; *P* = 0.7690; ANOVA test) (Fig. 1B, C, and 2B).

At day 3, significantly larger colonies of L. pneumophila were obtained in the medium manufactured without O_2_: 1.51 ± 0.098 mm versus 0.84 ± 0.09 mm; *P* < 0.001; Student’s *t* test for independent data (spiral-pour method), and 1.01 ± 0.067 mm versus 0.51 ± 0.035 mm; *P* < 0.001; Student’s *t* test for independent data (membrane filtration) (Fig. 3B).

As L. anisa is associated with a slower growth, leading to a more difficult detection, the faster growth observed in the medium produced without O_2_ is of particular interest. Notably, at day 3, colonies were only detected in the medium produced by autoclaving at 115°C for 15 min without O_2_ and using the spiral-pour method: the mean number of CFU was 59 ± 6.36 and the mean size of colonies was 0.88 ± 0.062 mm (Fig. 1E, 2B, and 3B).

At day 5, L. anisa also showed improved growth in the medium manufactured without O_2_ removal, using the spiral-pour method: 88.09 ± 8.10 CFU versus 73.25 ± 7.89 CFU; *P* < 0.05; ANOVA test, whereas membrane filtration resulted in similar counts (42.48 ± 6.04 CFU versus 44.00 ± 4.71 CFU; *P* = 0.3039; ANOVA test) (Fig. 1E and 2). Accordingly, a higher mean size of colonies was also observed with the spiral-pour method (2.7 ± 0.23 mm versus 1.16 ± 0.24 mm; *P* < 0.001; Student’s *t* test for independent data) and after membrane filtration: 1.71 ± 0.19 mm versus 1.17 ± 0.13 mm; *P* < 0.001 (Fig. 1E and 3B).

Demonstrating the reproducibility of the modified method, similar results were obtained on different days (variation coefficients from 3.9% to 14.2%; from 2 to 8 different days for each comparison).

Regarding selectivity properties, the medium produced without O_2_ and with modified autoclaving conditions exhibited higher selectivity properties than the medium with modified autoclaving alone, particularly for Enterococcus faecalis ATCC 29212, which was completely inhibited in the absence of O_2_, yet grew in the other conditions (> 1,000 CFU). A trend toward a higher selectivity for P. aeruginosa was also observed (0.37 log reduction) (Table 1; Fig. 4).

**TABLE 1:** Selectivity results and comparison of selectivity properties of GVPC manufactured in different conditions at day 3

GVPC medium under different conditions	Mean no. of colonies			
	P. aeruginosa ATCC 9027	E. faecalis ATCC 29212	E. faecalis ATCC 19433	E. coli ATCC 25922
Conditions 1: 121°C, 20 min - O_2_ presence - N_2_ absence - Presence of activated carbon	>1,000	>1,000	0	0
Conditions 2: 115°C, 15 min - O_2_ presence - N_2_ absence - Presence of activated carbon	>1,000	>1,000	0	0
Conditions 3: 115°C, 15 min - O_2_ absence - N_2_ absence - Presence of activated carbon	87.75 ± 13.86	0	0	0
Conditions 4: 115°C, 15 min - O_2_ absence - N_2_ presence - Presence of activated carbon	5.33 ± 2.75	0	0	0
Conditions 5: 115°C, 15 min - O_2_ presence - N_2_ absence - Absence of activated carbon	0	>1,000	0	0
Conditions 6: 115°C, 15 min - O_2_ absence - N_2_ absence - Absence of activated carbon	0	0	0	0

### Effect of N_2_ injection together with O_2_ removal during ingredient stirring versus the effect of O_2_ removal alone (conditions 4 versus conditions 3).

**FIG 5:**
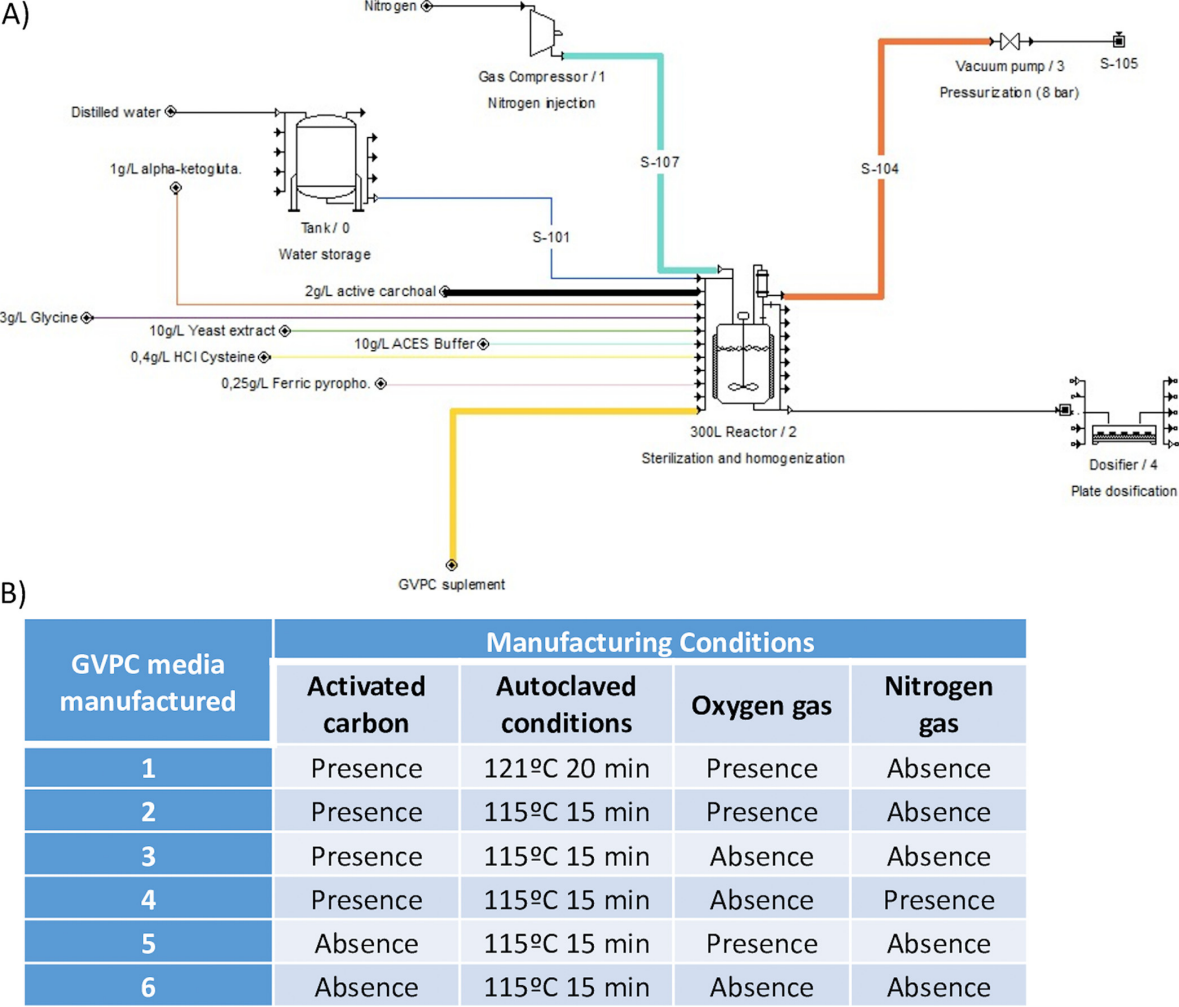
Bacterial growth of *L. pnemophila* and L. anisa after spiral pouring on GVPC manufactured in different conditions: 115°C, 15 min, O_2_ removal and nitrogen injection (conditions 4) versus 115°C, 15 min, without O_2_ (conditions 3) versus modified autoclaved conditions alone (115°C, 15 min; conditions 2).

The injection of N_2_, together with O_2_ removal and modified autoclaving, yielded the best results in terms of *Legionella* growth. With the improved conditions, colonies were visible significantly earlier: at day 2 for L. pneumophila and day 3 for L. anisa, with higher sizes than when using the previously assessed conditions. In comparison with conditions 3, the injection of N_2_ produced statistically significant improvements in the number of CFU and colony size at days 2 and 3 for L. pneumophila; at day 2, a 28.9% increase was obtained in the mean number of colonies of L. pneumophila using the spiral-pour method, without statistically significant differences: 158 ± 38.88 CFU versus 99.66 ± 20.98 CFU; (*P* = 0.1861, ANOVA test). Accordingly, higher colony sizes were obtained in the medium manufactured with N_2_ injection: 0.69 ± 0.049 mm versus 0.41 ± 0.027 mm; *P* < 0.001 (Fig. 2C, 3C, and 5).

At day 3, similar CFU were obtained in the media produced by N_2_ injection or O_2_ removal alone (88.81 ± 4.82 CFU versus 94.25 ± 4.12 CFU; *P* = 0.2658; ANOVA test), although the former yielded higher colony sizes: 1.75 ± 0.122 mm versus 1.37 ± 0.107 mm; *P* < 0.001; Student’s *t* test for independent data (Fig. 2C, 3C, and 5). After membrane filtration, at day 3, colony growth was very similar between conditions 4 versus conditions 3: 70.26 ± 7.80 CFU versus 70.375 ± 8.02 CFU; *P* = 0.2658, with mean colony sizes of 1.04 mm versus 1.00 mm (*P* = 0.348 Student’s *t* test for independent data) (Fig. 3C).

In the case of L. anisa, colonies were visible at day 3 in both media: after N_2_ injection, 36 CFU were obtained versus 50 CFU and 0.99 ± 0.072 mm versus 0.74 ± 0.05 mm (*P* < 0.001). Higher numbers of colonies were also determined by the spiral-pour method at day 5 after N_2_ injection, with statistically significant differences (56.57 ± 2.82 CFU versus 50.75 ± 5.95 CFU; *P* = 0.0392; ANOVA test), whereas the results were similar after membrane filtration (33.95 ± 5.30 CFU versus 30.75 ± 3.62 CFU, *P* = 0.2175), with colony sizes of 2.23 ± 0.12 mm versus 2.42 ± 0.145 mm (*P* = 0.040) and 1.23 ± 0.09 versus 1.33 ± 0.068 mm (*P* = 0.075), respectively (Fig. 2C, 3C, and 5). In comparison with the medium modified only in the autoclaving conditions (conditions 2), a more pronounced improvement in all growth markers (CFU, colony size) was found in both L. pneumophila (day 3) and L. anisa (day 5) (Fig. 1B, C, 2D, and 3D).

Demonstrating the reproducibility of the method, similar results were obtained at different days (variation coefficients ranging from 2.7% to 11.1%; from 2 to 8 different days for each comparison).

The GVPC medium manufactured without O_2_ in the presence of N_2_ and autoclaved at 115°C for 15 min showed improved selectivity properties, particularly for P. aeruginosa and E. faecalis strain ATCC 29212. The medium prepared with N_2_ completely inhibited the Enterococcus faecalis strain ATCC 29212 (0 CFU) and also E. faecalis ATCC 19433 and E. coli ATCC 25922 (Table 1; Fig. 3), and resulted in the complete or partial inhibition of P. aeruginosa growth (5.33 CFU versus 88 CFU; *p* <0.001) (Fig. 4; Table 1).

In comparison with other marketed GVPC media, the medium manufactured with N_2_ also exhibited higher selectivity properties, particularly for E. faecalis ATCC 29212 and P. aeruginosa.

### Effect of charcoal removal (conditions 5 and conditions 6).

Using the modified medium with improved properties, activated charcoal was removed to test if its absence would also allow the growth of *Legionella* and inhibit the selectivity strains.

In the absence of activated charcoal, GVPC autoclaved at 115°C for 15 min prevented the growth of both L. pneumophila (at day 3) and L. anisa (at day 5) determined by the spiral-pour method and after membrane filtration. In the selectivity assays, the medium without activated charcoal allowed the growth of E. faecalis 29212 (> 1,000 CFU) and completely inhibited E. faecalis ATCC 19433, E. coli ATCC 25922, and P. aeruginosa ATCC 9027.

In the absence of activated charcoal, GVPC manufactured without O_2_ and autoclaved at 115°C for 15 min prevented the growth of L. pneumophila (direct plating and after membrane filtration). In the case of L. anisa, growth was only observed after membrane filtration (59 CFU at day 5). In the selectivity assays, complete inhibition of growth was observed for E. faecalis (both strains ATCC 29212 and ATCC 19433), E. coli (ATCC 25922), and P. aeruginosa (ATCC 9027).

### Productivity and selectivity properties of the improved medium using real water samples.

GVPC manufactured without O_2_ and autoclaved at 115°C for 15 min also exhibited improved selectivity properties, inhibiting the growth of interfering microorganisms in real water samples obtained from cooling towers, in comparison with the medium manufactured with the same conditions but with O_2_.

The significant reduction in growth of interfering microorganisms (medium without O_2_ versus medium with O_2_) was observed after filtration and filter resuspension (15.00 ± 1.41 CFU versus 30.00 ± 5.65 CFU), heat treatment (4.00 ± 2.82 CFU versus 22.00 ± 2.82 CFU) and acid treatment (7.00 ± 2.82 CFU versus 32.50 ± 6.36 CFU) (Table 2).

**TABLE 2:** Analysis of productivity and selectivity properties of GVPC manufactured without O_2_ and autoclaved at 115°C, 15 min (conditions 3 versus conditions 1) using real samples from cooling towers^*a*^

Type of sample	Phase^*b*^	GVPC Condition 1	GVPC Condition 3
		Interfering microorganisms (CFU/plate)	Interfering microorganisms (CFU/plate)
Water sample n°1	Phase A	0	0
	Phase B	30.00 ± 5.65	15.00 ± 1.41
	Phase C	22.00 ± 2.82	4.00 ± 2.82
	Phase D	32.50 ± 6.36	7.00 ± 2.82
Water sample n°1 + *Legionella* inoculation	Phase A	1.50 ± 0.70	0
	Phase B	34.50 ± 20.50	5.00 ± 2.82
	Phase C	0	0
	Phase D	17.00 ± 5.65	1
Water sample n°2	Phase A	0	0
	Phase B	5.50 ± 0.70	0
	Phase C	5.00	0
	Phase D	6.50 ± 3.53	0
Water sample n°2 + *Legionella* inoculation	Phase A	0	0
	Phase B	10.50 ± 0.70	0
	Phase C	7.50 ± 2.12	0
	Phase D	9.00 ± 1.41	0

a Colony count (CFU/plate) of interfering microorganisms.

b Phase A, direct plating; phase B, plating of concentrated water from filtration; phase C, heat treatment (50°C, 30 min); phase D, acid treatment (HCl-ClK buffer, pH 2.2. for 5 min). The colony reading was performed after 5 incubation days at 37°C.

After filter resuspension, in samples contaminated with L. pneumophila and L. anisa, *Legionella* concentrations were 300 CFU/L using the medium without O_2_ and 200 CFU/L with the medium manufactured in normal conditions (Table 3).

**TABLE 3:** Analysis of productivity properties of GVPC manufactured without O2 and autoclaved at 115°C, 15 min (conditions 3 vs conditions 1) using real samples from cooling towers^*a*^

Type of sample	Phase^*b*^	CFU/L *Legionell*a spp.	
		GVPC Condition 1	GVPC Condition 3
Water sample n°1	Phase A	0	0
	Phase B	100	50
	Phase C	0	0
	Phase D	50	0
Water sample n°1 + *Legionella* inoculation	Phase A	0	0
	Phase B	100	50
	Phase C	0	0
	Phase D	0	0
Water sample n°2	Phase A	0	0
	Phase B	0	50
	Phase C	0	0
	Phase D	0	0
Water sample n°2 + *Legionella* inoculation	Phase A	0	50
	Phase B	200	300
	Phase C	50	0
	Phase D	50	0

a *Legionella* colony count (CFU/L) of water samples from cooling waters.

b Phase A, direct plating; Phase B, plating of concentrated water from filtration; Phase C, heat treatment (50°C, 30 min); Phase D, acid treatment (HCl-ClK buffer, pH 2.2. for 5 min). The colony reading was performed after 5 incubation days at 37°C.

### Analyses at the end of shelf life.

Analysis of results showed that the productivity and selectivity of the manufactured media were maintained until the end of shelf life (3 months). Similar CFU values were obtained for L. pneumophila and L. anisa seeded by the spiral-pour method and membrane filtration, and complete inhibition was observed at both time 0 and 3 months for E. coli, E. faecalis ATCC 19433, and ATCC 29212. Partial inhibition of P. aeruginosa was observed at both time points (22 CFU at time 0 and 24 CFU at the end of shelf life).

## DISCUSSION

The quality of the culture media has considerable impact on the recovery of *Legionella* spp. (25), and may complicate the interpretation and comparison of results obtained in different laboratories (26).

The currently available media render *Legionella* detection in water samples a challenging task, as they have a low differential capacity and only partially inhibit interfering microorganisms that grow faster than *Legionella.* For this reason, increasing the *Legionella* growth rate and improving the selectivity properties of the medium is of vital importance, particularly in media with a high or very high concentration of interfering microorganisms, such as P. aeruginosa. For the analysis of water samples, GVPC, MWY, or BCYE-AB are the media recommended by the new edition of ISO 11731:2017 (16, 22).

The aim of the present study was therefore to find simple modifications in GVPC manufacture that could improve both the growth of *Legionella* spp. and selectivity properties. The first modification introduced was to moderate the autoclaving conditions by reducing the temperature by 6°C and duration by 5 min, which yielded better results in terms of L. pneumophila and L. anisa growth, an improvement that was further enhanced by O_2_ removal during the stirring of ingredients. This initial improvement could be attributed to a lower generation of hydrogen peroxide and superoxide radicals during autoclaving, to which *Legionella* species are particularly sensitive (20, 21). According to previous studies, autooxidation and photochemical oxidation of the components of such a complex media, including yeast extract, during autoclaving would accelerate the production of O_2_ radicals (20, 27). As previously reported, L. pneumophila does not grow in autoclaved yeast extract medium in the absence of charcoal (20, 26), unless it is sterilized by filtration (20, 28). The importance of reducing the formation of reactive oxygen species (ROS) during autoclaving was also demonstrated in a recent study of a medium designed for the growth of environmental bacteria, in which ROS generation was minimized by separately autoclaving the phosphate and agar, thereby improving the culturability of microorganisms (29).

Instead of ingredient separation, modifying the autoclaving conditions is a more feasible strategy to avoid ROS in GVPC manufacture, saving both time and energy. Autoclaving adjustments to improve medium properties are also accepted by ISO 11133:2014 (30), according to which the sterilization cycle of the autoclave can be adapted to high volumes to ensure adequate heat treatment.

In the new autoclaving conditions, O_2_ removal during GVPC manufacture using a vacuum pump system and N_2_ injection were instrumental in enhancing the growth rate of both L. pneumophila and L. anisa. These results could also be explained by the reduced oxidation of key ingredients, such as yeast extract, during the stirring, which led to a significant decrease in the ROS concentration in the final medium.

In the *Legionella* genus, as the reduced-oxygen-scavenging enzymes are not uniformly present (20, 21, 26), certain species are especially sensitive to ROS (20). In the case of L. pneumophila, two bifunctional catalase-peroxidases (KatA and KatB) have been described (31). Nevertheless, neither KatA nor KatB are essential for exponential growth in complex media in aerobic conditions (32) and both enzymes are more active in the stationary phase of growth (conditions that favor the transition of replicating bacteria into a transmissible form) and during intracellular multiplication (33).

A notable finding in our study was the earlier growth observed in L. pneumophila at day 2, and in L. anisa at day 3, which was correlated with the confluence of different factors, such as the autoclaving conditions, and O_2_ removal with the vacuum pump system and N_2_ injection. Another relevant finding is the earlier growth of L. anisa, with colonies being observed for the first time, at day 3. L. anisa, together with L. pneumophila, is included in the productivity quality control of GVPC medium (ISO 11731:2017) (16), being characterized to have slower growth than L. pneumophila (24). A faster growth of *Legionella* spp. in the improved medium is a relevant result that may facilitate an earlier recovery in real samples. This constitutes a significant improvement in *Legionella* detection, for both L. pneumophila and L. anisa.

Detection of L. anisa in GVPC is important, as it is the most common non*-pneumophila Legionella* species in the environment, can be a causative agent of legionellosis and Pontiac fever, and may be hospital-acquired (34, 35). Epidemiological studies show that the homes of approximately 20% of Legionnaires’ disease patients tested positive for *Legionella* (mostly L. anisa) (36, 37). In addition, this bacterium has been found to cause extra-pulmonary infections such as chronic endocarditis (35, 38) and osteomielitis (35).

Having achieved positive effects on *Legionella* growth by modified autoclaving conditions and O_2_ removal, we tested the removal of activated charcoal. However, using these conditions, growth of L. pneumophila in GVPC was not possible and only L. anisa growth was determined after membrane filtration, thus confirming the key role of activated charcoal in GVPC as a scavenger of radicals and peroxides (21, 22).

Further research is needed to find the conditions that would allow the removal of activated charcoal, which could lead to the development of a differential and even chromogenic medium, without the inconvenience of its black appearance.

Another relevant finding of this study are the enhanced selectivity properties of the medium autoclaved at 115°C for 15 min, with O_2_ vacuum removal and N_2_ injection. With these conditions, complete inhibition of both strains of E. faecalis (ATCC 29212 and ATCC 19433) and E. coli was achieved, with complete or partial inhibition of P. aeruginosa.

Strong selectivity properties of GVPC are essential for water samples with high concentrations of interfering microorganisms. In our study, the modified GVPC medium was selective in real water samples from cooling towers, without reducing the recovery of *Legionella* spp. due to possible contaminating microbiota (22). Nevertheless, the modified media needs further evaluation in real samples.

The enhanced selectivity for P. aeruginosa is particularly relevant, as the species was eliminated as a criterium for this medium in ISO 11731:2017. Selectivity for P. aeruginosa is vital in the analysis of samples from different types of water systems that suffer from frequent contamination by *Legionella* and P. aeruginosa (39, 40), including biofilms associated with plumbing systems (40).

The observed inhibition of P. aeruginosa in the medium manufactured with less O_2_ may be attributed to a possible interaction between activated charcoal and polymyxin B. We have demonstrated that this strain (ATCC 9027) is sensitive to this antibiotic, but it is always inhibited in the absence of charcoal. We postulate that in GVPC, polymyxin B, a lipopeptide consisting of a polycationic peptide ring and a tripeptide side chain (41, 42), would interact through its positive charges with the high negatively charged surface area of activated charcoal, which, in the presence of O_2_, can have more negative groups, such as -COO- and -O- (43). As a result, polymyxin B becomes less active against P. aeruginosa. However, if O_2_ is removed, the antibiotic would not interact with the -COO- and -O- groups of the charcoal and would be more effective against P. aeruginosa (43). More studies are required to fully characterize this interaction, considering previous reports of the capacity of charcoal to remove different types of antibiotics through high adsorption (44) and charcoal-oxygen-nitrogen interactions (43, 45). Another possible explanation could be related to the secondary mechanism of action of polymyxin B, in which an oxidative burst is generated when it interacts with P. aeruginosa (46). The effect of a decrease in ROS in the culture medium and the presence of less O_2_ and more N_2_ during mixing on the antibiotic activity of polymyxin B should be clarified in future studies.

Another interesting finding of this study is the increased selectivity of GVPC for E. faecalis, particularly for strain ATCC 29212, which, despite being one of the E. faecalis strains recommended by ISO11133:2017, is usually not used for the quality control of GVPC media. Based on these results, both E. faecalis strains recommended by ISO11731:2017 could be used (ATCC 19433, equivalent to WDCM 00009 and ATCC 29212, equivalent to WDCM 00087). The absence of inhibition in GVPC could also be attributed to interactions of the antibiotic with activated charcoal in the presence of O_2_, although the hypothesis is less plausible considering the glycopeptide vancomycin structure (47).

As inhibition of E. faecalis ATCC 29212 was observed only in the absence of O_2_, even without activated charcoal, other hypotheses could be postulated. For example, presence of O_2_ during medium preparation and the consequent ROS generation are two described factors that could contribute to vancomycin partial degradation (48), thus allowing growth of the more resistant strain (E. faecalis ATCC 29212).

In summary, we have demonstrated for the first time that more moderate autoclaving conditions (115°C, 15 min) and O_2_ removal with a vacuum pump system significantly enhances GVPC quality, in terms of both productivity and selectivity. The effect of N_2_ injection still requires further research. Oxygen removal during GVPC manufacture increased the growth of L. pneumophila and L. anisa, and markedly inhibited the growth of E. coli, P. aeruginosa, and E. faecalis. We consider that these simple modifications are easy to implement, and in the case of the autoclaving conditions, save time and energy.

## MATERIALS AND METHODS

### Manufacturing GVPC medium in different conditions.

**FIG 6:**
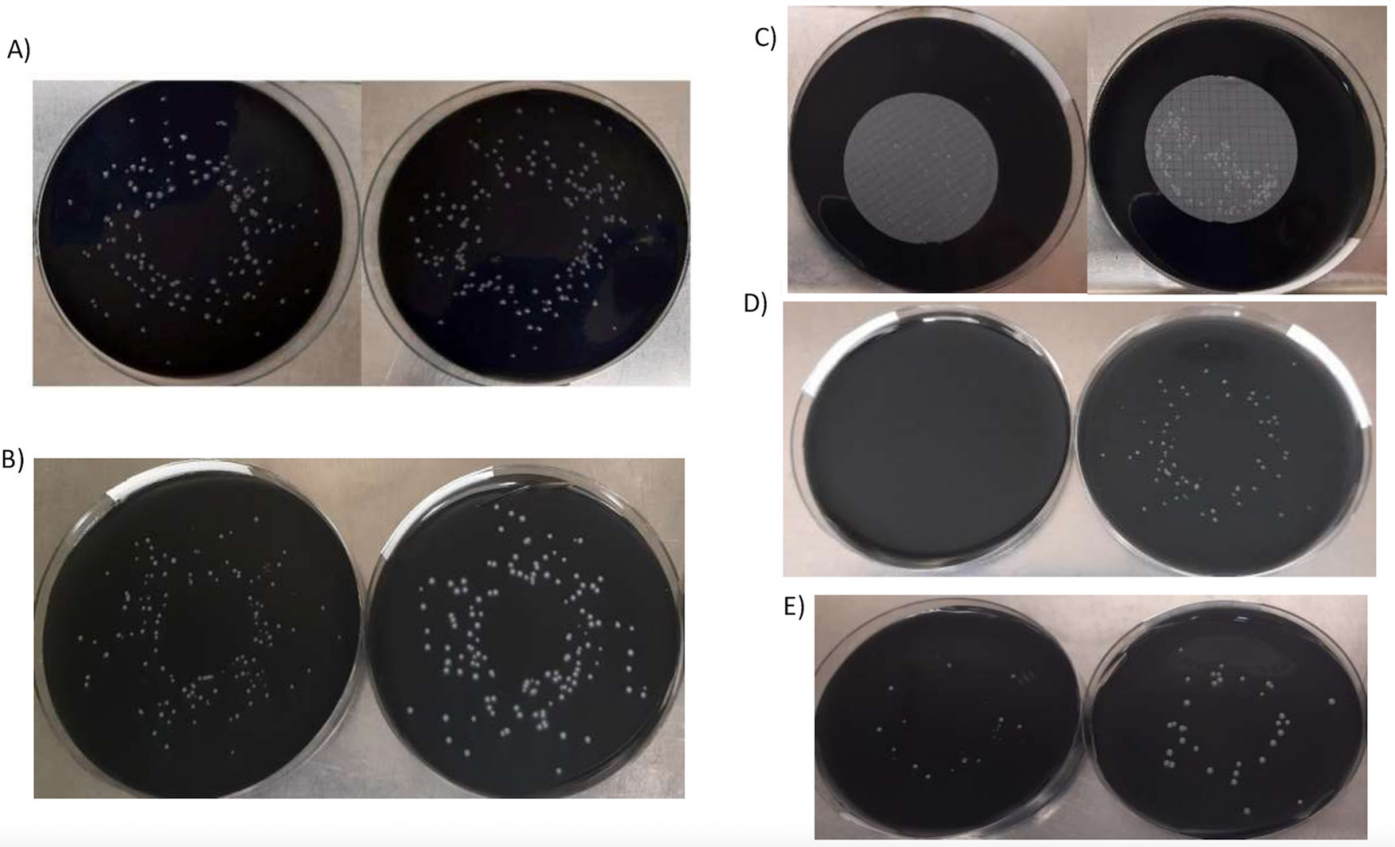
(A) Flow chart of the manufacturing process of GVPC in different conditions. (B) GVPC manufactured in different conditions.

GVPC agar was manufactured at Reactivos para Diagnóstico, S.L. (Sentmenat, Barcelona, Spain) using different conditions, based on modification of autoclaving conditions, O_2_ removal and N_2_ injection during medium manufacturing, and charcoal removal. In total, six different conditions were evaluated in this study: (i) normal conditions (presence of O_2_, autoclaving at 121°C, 20 min); (ii) presence of O_2_, autoclaving at 115°C, 15 min; (iii) absence of O_2_, autoclaving at 115°C, 15 min; (iv) absence of O_2_, autoclaving at 115°C, 15 min and injection of N_2_; (v) presence of O_2_, autoclaving at 115°C, 15 min in the absence of charcoal; (vi) absence of O_2_, autoclaving at 115°C, 15 min without charcoal (Fig. 6).

GVPC was manufactured on a pilot scale for this study (see flow chart in Fig. 6). In normal conditions (condition 1), autoclaving of ingredients was performed at 121°C for 20 min, with the later addition of membrane filtered supplements (Fig. 6).

In the second set of conditions, medium manufacture was identical except for the autoclaving, which was carried out at 115°C for 15 min. These modifications were also applied to conditions 3, 4, and 5. In condition 3, O_2_ was partially removed during the stirring of ingredients using a liquid ring vacuum pump system connected to the reactor and with recirculation in the addition of activated charcoal to avoid precipitation. Oxygen removal was also applied in the autoclaving, cooling and dispensing processes.

To produce a more marked decrease in the concentration of O_2_ during GVPC production, continuous N_2_ injection (purity 99.99%, Carburos Metálicos, SA, Barcelona, Spain) was also introduced during ingredient stirring in the reactor connected to the vacuum pump (condition 4). Finally, GVPC was also manufactured without activated charcoal, in the presence or absence of O_2_ in the reactor and autoclaved at 115°C for 15 min (conditions 5 and 6).

For all the conditions, the final mixture was dispensed in 20 mL volumes into Petri dishes 90-mm diameter in a cleanroom. Plates were stored in airtight containers.

### Inocula preparation.

Inocula were prepared according to ISO11133:2014/amended 1:2018 (30). All tested bacterial strains were obtained directly from the reference culture collection (American Type Culture Collection [ATCC]). A single subculture from the reference strains was used to obtain reference stock strains from which stock and working cultures were prepared using non-selective media: TSA slant tubes for E. faecalis strains, P. aeruginosa, E. coli, and BCYE for *Legionella* strains. Each strain was verified before use in the appropriate selective and differential media according to ISO 17025:2017 (49).

Stock dilutions were prepared according to ISO 11731:2017 (16) and ISO 8199:2018 (50) and adjusted using turbidimetry (McFarland unit of 5 for productivity and McFarland unit of 2 for selectivity assays) (Densitometer DEN-1B, Grant instruments, Cambridgeshire, UK), from which serial dilutions were prepared in the same diluent. For the productivity assays (with *Legionella* species), serial dilutions were prepared to obtain 50 to 120 CFU per plate. For the selectivity assays (with P. aeruginosa, E. faecalis, and E. coli), serial dilutions were prepared to obtain ≥10^4^ CFU per plate, according to ISO 11133:2014/amended 1:2018.

The strains used were those recommended by ISO 11731:2017: Enterococcus faecalis ATCC 19433 and 29212 (equivalent to WDCM 00009 and 00087, respectively) and E. coli ATCC 25922 (equivalent to WDCM 00013). P. aeruginosa 9027 (equivalent to WDCM 00026) was used in the selectivity assays.

Samples of 100 μL were plated onto GVPC medium by the spiral-pour method (Eddyjet, IULmicro, Barcelona, Spain). In the productivity assays, plating was also performed after filtration with 0.45 μm pore mixed cellulose ester membrane filters of a bacterial suspension in a diluent containing < 80 CFU of *Legionella* species, according to ISO 8199:2018 (23).

In the productivity assays, plates were incubated at 36 ± 2°C for periods of 2 to 5 days for L. pneumophila and 5 to 10 days for L. anisa. In the selectivity assays, plates were incubated at 36 ± 2°C for 3 days. The number and size of colonies were measured at days 2, 3, and 5 in the productivity assays and at day 3 in the selectivity assays.

### Colony count.

The colony count was performed using an automatic colony counter (SphereFlash, IULmicro, Barcelona, Spain). The size of colonies was obtained first in pixels and then converted to millimeters using the automatic colony counter and also the mobile application Pixel Measure 1.0 (Leroy Hopson Apps, Vietnam).

### Productivity assays.

Productivity assays were performed according to ISO 11731:2017 (16), with the following strains: L. pneumophila (ATCC 33152; WDCM 00107) and L. anisa (ATCC 35292; WDCM 00106).

Colonies of *Legionella* generally appeared white-gray, but other colors could appear. Colonies were smooth with an entire edge and had a characteristic ground-glass appearance.

### Selectivity assays.

Selectivity assays were performed according to ISO 11731:2017 (16) and ISO 11133:2014/amd 1: 2018 (30), with the following strains: E. faecalis (ATCC 19433, equivalent to WDCM 00009 and ATCC 29212, equivalent to WDCM 00087), and E. coli (ATCC 25922 equivalent to WDCM 00013). P. aeruginosa (ATCC 9027 equivalent to WDCM 00026) was also tested.

### Collection of water samples.

Water samples from two evaporative condensers were kindly provided by Eminfor S.L. (Barcelona, Spain). To test the quality of the media, the water samples were analyzed using two different types of GVPC medium: GVPC manufactured in normal conditions (conditions 1) and GVPC manufactured with O_2_ removal and autoclaved at 115°C for 15 min without N_2_ injection (conditions 3).

Water samples of 1L were collected aseptically in sterile containers containing a neutralizing agent (sodium thiosulfate) and transported to the laboratory, where they were stored at 5 ± 3°C until needed. For each sample, two aliquots (500 mL) were analyzed: direct samples and laboratory samples contaminated with L. pneumophila (500 to 1,000 CFU/L) and L. anisa (500 to 1,000 CFU/L).

Samples were prepared according to ISO11731:2017 (16) for samples with a high concentration of interfering microorganisms: direct plating, plating of concentrated water from filtration, and heat treatment (50°C, 30 min) and acid treatment (HCl-ClK buffer, pH 2.2. for 5 min).

Two aliquots of 100 μL of each portion were plated onto GVPC (manufactured in normal conditions [conditions 1]) and modified autoclaving in the absence of O_2_ (conditions 3).

The plates were incubated at 36 ± 2°C for 10 days and were inspected for the first time on day 2 and manually read every 2 days until the end of the incubation period.

### Analyses of the media at the end of shelf life.

Productivity and selectivity controls were performed after manufacturing and at the end of shelf life (3 months) for the medium manufactured without O_2_ and autoclaved at 115°C for 15 min (conditions 3).

### Statistical analysis.

Each sample was plated in duplicate on all direct plating media. All data were analyzed by the general linear model form using SPSS v.21.0 (IBM Corp., Chicago, IL, USA). The mean and standard deviation were calculated for all measures (number of colonies/plate, size of colonies). The numbers of CFU obtained in media manufactured with different methods were subjected to analysis of variance (ANOVA test) using the general linear model procedure to eliminate inter-day variability. The number of bacterial colonies growing on an agar plate was presumed to follow a Poisson distribution, so the square root was extracted to normalize the data and to apply the ANOVA tests.

The size of colonies (mm) grown in different media was compared using the Student’s *t* test for independent data. In all tests, the significance level alpha was set as 0.05.
